# Which insulin resistance-based definition of metabolic syndrome has superior diagnostic value in detection of poor health-related quality of life? Cross-sectional findings from Tehran Lipid and Glucose Study

**DOI:** 10.1186/s12955-015-0391-5

**Published:** 2015-12-09

**Authors:** Tina Deihim, Parisa Amiri, Reza Taherian, Maryam Tohidi, Asghar Ghasemi, Leila Cheraghi, Fereidoun Azizi

**Affiliations:** Research Center for Social Determinants of Endocrine Health, Research Institute for Endocrine Sciences, Shahid Beheshti University of Medical Sciences, Tehran, Iran; Students’ Research Committee, Shahid Beheshti University of Medical Sciences, Tehran, Iran; Prevention of Metabolic Disorders Research Center, Research Institute for Endocrine Sciences, Shahid Beheshti University of Medical Sciences, Tehran, Iran; Endocrine Physiology Research Center, Research Institute for Endocrine Sciences, Shahid Beheshti University of Medical Sciences, Tehran, Iran; Endocrine Research Center, Research Institute for Endocrine Sciences, Shahid Beheshti University of Medical Sciences, Tehran, Iran

**Keywords:** Metabolic syndrome, Health-related quality of life, Insulin resistance

## Abstract

**Background:**

The superiority of the diagnostic power of different definitions of metabolic syndrome (MetS) in detecting objective and subjective cardiovascular outcomes is under debate. We sought to compare diagnostic values of different insulin resistance (IR)-based definitions of MetS in detecting poor health-related quality of life (HRQoL) in a large sample of Tehranian adults.

**Methods:**

This cross-sectional study conducted within the framework of the Tehran Lipid and Glucose Study on a total sample of 742 individuals, aged ≥ 20 years. Metabolic syndrome was defined according to the World Health Organization (WHO), the European Group for the study of Insulin Resistance (EGIR), and the American Association of Clinical Endocrinology (AACE). Health-related quality of life was assessed using the Short Form Health Survey (SF-36). Logistic regression analysis and Receiver Operating Characteristic (ROC) curve were used to investigate the impact of the three IR-based definitions of MetS on HRQoL and compare their discriminative powers in predicting poor HRQoL.

**Results:**

Compared with other definitions, the WHO definition identified more participants with MetS (41.8 %). Although the AACE definition had higher adjusted odds ratios for reporting poor physical HRQoL (OR: 1.95; CI: 0.84–4.53 and OR: 1.01; CI: 0.55–1.85 in men and women respectively) and mental HRQoL (OR: 0.97; CI: 0.41–2.28 and OR: 1.00; CI: 0.56–1.79 in men and women respectively), none of the three studied definitions were significantly associated with poor physical or mental HRQoL in either gender; nor did ROC curves show any significant difference in the discriminative powers of IR-based definitions in detecting poor HRQoL in either gender.

**Conclusions:**

None of the three studied IR-based definitions of MetS could significantly detect poor HRQoL in the physical or mental domains, indicating no significant superior diagnostic value for any of these definitions.

## Background

Metabolic syndrome (MetS), an escalating health issue worldwide, is a constellation of metabolic abnormalities, which are the major risk factors for developing cardiovascular disease (CVD) and diabetes type 2 [1]. Fast increasing evidence shows the negative effect of MetS on health-related quality of life (HRQoL) as a subjective patient-centered health measurement that concentrates on the individual's own perception of their health status and life satisfaction [2]. Recent data reveals that the prevalence of the syndrome is fast increasing in different populations [3] and is reported to be approximately 34.7 % in Iranian adults [4].

Despite much research have been conducted in recent years, there is uncertainty regarding the concept of MetS and critical investigations have questioned whether the syndrome is a mere aggregation of metabolic abnormalities or a syndrome representing a clinical entity [5]. This doubt has resulted in the introduction of several definitions that include risk factor components which are not entirely similar and more importantly, the cut-offs used for defining them differ. Insulin resistance (IR); the most accepted pathophysiology of MetS, is likely a significant link between the components of this syndrome [1]. On the other hand, obesity, which is included in most definitions of MetS, is identified as the most important correlate of the increasing prevalence of MetS [1]; in addition it has been proposed that IR is significantly related to central obesity [6]. Among the proposed definitions, the World Health Organization (WHO) [7], the European Group for the study of Insulin Resistance (EGIR) [8], and the American Association of Clinical Endocrinology (AACE) [9] emphasize IR as the major component of MetS; however, the National Cholesterol Education Program Adult Treatment Panel III (NCEP-ATP III), the American Heart Association/National Heart, Lung, and Blood Institute (AHA/NHLBI), the International Diabetes Federation (IDF) and the joint interim statement (JIS), emphasize waist circumference (WC) [10].

Considering the above mentioned ambiguity, several efforts have been made to explore the superior diagnostic powers of the WC- and IR-based definitions of MetS in detecting objective and subjective cardiovascular outcomes, i.e. CVD and HRQoL. Regarding objective CVD complications, evidence shows that except for the EGIR definition, the NCEP/AHA, AACE, IDF and modified WHO definitions are all associated with cardiovascular events among an elderly American population [11]. The results of a recent study of the Tehran lipid and glucose study (TLGS) population revealed no differences among diagnostic values of WC-based definitions of MetS in detecting coronary heart disease (CHD) and CVD [12]. Furthermore, in a Dutch population, compared to the WC-based definitions of MetS, IR-based definitions had lower hazards ratios in detecting cardiovascular events [13]. In the field of subjective outcomes, the association between WC-based definitions of MetS and HRQoL, especially in the physical domain and mainly in women has been reported [14]. We recently showed that these definitions failed to show any superiority in the discriminative powers in detecting poor HRQoL in Tehranian adults without diabetes [15]. Although the association between IR and HRQoL has been documented [16] there is no study reporting the association between IR-based definitions of MetS and HRQoL. As one of the first efforts, this study aimed to investigate diagnostic values of three IR-based definitions of MetS including WHO, EGIR and AACE to detect poor HRQoL in a large population of Tehranian adults.

## Methods

### Subjects and design

The current study was conducted within the framework of the TLGS, a large scale ongoing community based prospective study being performed on a representative sample of residents of district-13 of Tehran, the capital of Iran. Details of the rationale and design of the TLGS have been published elsewhere [17]. The TLGS has two major components: 1) a cross-sectional prevalence study of non-communicable diseases and their associated risk factors implemented from 1999 to 2001 and 2) a prospective follow-up study in which non-communicable diseases risk factors are measured approximately every 3 years.

In the current study, a total of 742 individuals, aged ≥ 20 years, with insulin measurements, participating in the TLGS between September 2005 and September 2007 (the second follow-up), was recruited. Information data of these 742 participants was analyzed using the WHO definition. For EGIR and AACE definitions, after excluding 135 persons with diabetes and 17 persons with missing data, the information of 590 persons met the inclusion criteria and was analyzed. The study was approved by the ethics committee of the Research Institute for Endocrine Sciences, Shahid Beheshti University of Medical Sciences and written informed consent was obtained from all participants.

### HRQoL measures

To assess HRQoL, we used the Iranian version of Short Form Health survey (SF-36), which has been validated in Iran [18]; this widely used questionnaire contains 36 questions summarized into eight subscales; four physical health related subscales including physical functioning, role limitations due to physical health problems, bodily pain, general health and also four mental health-related subscales including vitality, social functioning, role limitations due to emotional problems, and mental health. The physical subscales are summarized and termed as the physical component summary (PCS) and similarly the four mental subscales are termed as the mental component summary (MCS) [19]. The score attributed to each subscale ranges from 0 to 100 as the worst and the best conditions of health respectively. Calculating of the PCS and the MCS scores was done using the Quality Metric Health Outcomes Scoring Software 2 [20].

### Definitions

Metabolic syndrome was defined according to the WHO [7], EGIR [8] and AACE [9] criteria (Table 1). Based on the criteria of the American Diabetes Association (ADA), diabetes was defined as fasting plasma glucose ≥ 7 mmol/L, 2-h post 75 g glucose load ≥ 11.1 mmol/L or receiving antidiabetic medications [21]. Insulin resistance was defined as the upper quartile of insulin level of baseline population from the second follow-up of TLGS. After excluding patients with diabetes, those who had received educational intervention, and participants of the current study, the cut point for IR was calculated. The median [range] insulin concentration was 7.45 mU/ml [0.2–51.84] with an upper quartile range of ≥10.63. Menopause was defined as the time of cessation of menstrual periods for 12 consecutive months, not due to surgery or any other biological or physiological causes [22]. Impaired glucose regulation was defined according to the criteria of the ADA as fasting blood glucose 5.6 mmol/L to 6.9 mmol/L or 2-h post 75 g glucose load 7.8 mmol/L to 11.1 mmol/L [21]. Smoking status was considered in two groups: 1) Non- and ex-smokers and 2) Current smokers. Additional information regarding age, physical activity [23] and current use of oral hypoglycemic agents, lipid-lowering and anti-hypertensive medication were obtained using the TLGS data.

**Table 1:** Different definitions and their cut-offs used to define the metabolic syndrome

	World Health Organization (WHO)	European Group for the study of Insulin Resistance (EGIR)	American Association of Clinical Endocrinology (AACE)
Absolutely required	IGT^a^ or IFG^b^ or IR^c^ or diabetes	Plasma insulin > 75 percentile of persons without diabetes in the population under study.	High risk of being insulin resistant ^d^ (excluding diabetes)
Criteria	plus two or more of the below abnormalities	plus two or more of the four below abnormalities	Plus two or more of the four below abnormalities
Adiposity index	WHR > 0.90 in men and >0.85 in women and/or BMI > 30 kg/m2	WC ≥ 94 cm in men and ≥80 cm in women	none
Triglycerides	≥1.7 mmol/L and/or	>2 mmol/L and/or	>1.7 mmol/L
HDL	<0.9 mmol/L in men and <1.0 mmol/L in women	<1.0 mmol/L or treated for dyslipidemia	<1.0 mmol/L in men and <1.3 mmol/L in women.
Blood pressure	≥140/90 mmHg or treated for hypertension	≥140/90 mmHg or treated for hypertension	≥130/85 mmHg or treated for hypertension
Hyperglycemia	none	Fasting plasma glocuse ≥ 6.1 mmol/L (but without diabetes)	Fasting plasma glucose 6.1–6.9 mmol/L or IGT

*BMI*, body mass index; *WC*, waist circumference; *IGT*, impaired glucose tolerance; *IFG*, impaired fasting glucose; *HDL*, high density lipoprotein; *WHR*, waist to hip ratio ^a^120 min post glucose challenge (75 mg):7.8–11.1 mm/L ^b^Fasting plasma glucose 5.6–7.00 mm/L ^c^defined as plasma insulin > 75 percentile of persons without diabetes in the population under study ^d^For the AACE definition, high risk of being insulin resistant is indicated by the presence of at least one of the followings: diagnosis of hypertension or cardiovascular disease; family history of type 2 diabetes or history of gestational diabetes or glucose intolerance; BMI > 25 kg/m^2^ or WC > 94 cm (men), >80 cm (women); age >40 years

### Other measures

Waist circumference was measured at the umbilical level, over light clothing, using an unstretched tape meter, without any pressure to body surface and measurements were recorded to the nearest 0.1 cm. Blood pressure was measured twice, after participants were seated for 15 min, using a standard mercury sphygmomanometer; there was at least 30s interval between these two separate measurements and the mean of two measurements was recorded as the participants blood pressure. Twelve-hour fasting blood samples were collected in tubes containing 0.1 % EDTA and were centrifuged at 4 °C and 500 × g for 10 min, to separate the plasma. Blood glucose was measured on the day of blood collection by an enzymatic colorimetric method using glucose oxidase. Fasting serum insulin was determined by the electrochemiluminescence immunoassay (ECLIA) method, using Roche Diagnostic kits and the Roche/HitachiCobas e-411 analyzer (GmbH, Mannheim, Germany). Serum total cholesterol and triglyceride concentrations were measured with commercially available enzymatic reagents (Pars Azmoon, Tehran, Iran) adapted to a selectraautoanalyzer. High density lipoprotein-cholesterol (HDL-C) was measured after precipitation of the apolipoprotein B-containing lipoproteins with phosphotungistic acid. Low density lipoprotein-cholesterol was calculated from serum total cholesterol, triglycerides (TG), and HDL-C, except when TG concentration was > 4.5 mmol/L [17].

### Statistical analysis

By use of graphical methods, continuous variables were checked for normality and are expressed as mean ± SD. Distribution of variables with normal and non - normal distributions between two groups and also categorical variables were compared using sample *t*-test, Mann–Whitney test and *χ*2 test respectively; categorical variables are reported as percentages.

Poor HRQoL was defined as the first tertile of PCS or MCS and to compute the odds ratios (ORs), logistic regression analysis was used. Sex specific ORs with 95 % confidence intervals were computed for men and women separately; model 2 was adjusted for age (years) and model 3 was adjusted for age, smoking (only in men-Ref: never smoked or ex-smoker), education (Ref: above high school), menopause (only in women-Ref: productive age) and marital status (Ref: married). In women, smoking was not adjusted in model 3 because of the low number of smokers in this group. Statistical analysis was performed using SPSS software version 16 (SPSS Inc., Chicago, IL, USA), with significance set at p < 0.05. To evaluate the power of each of the three definitions of the MetS in predicting poor physical and mental HRQoL among the study population, the area under the curve (AUC) was computed for the fully adjusted model (model 3). Differences between areas under the receiver operating characteristic (ROC) curves for the different MetS definitions were tested using STATA software version 10 (STATA Inc., College Station, TX, USA).

## Results

General metabolic and clinical characteristics of study participants are listed in Table 2; compared to women, men had higher mean levels of WC, SBP, DBP (*P* < 0.001), but lower HDL-C (P < 0.001); compared to women, rates of higher education and married subjects were significantly higher in men (P <0.001). According to different MetS definitions, more participants (41.8 %) met the WHO definition, followed by AACE (30.7 %) and EGIR (25.6 %) criteria.

**Table 2:** General characteristics of study participants

	All (*n* = 742)	Men (*n* = 246)	Women (*n* = 496)	P-value
Age (y)	49.78 (13.25)	51.25 (14.13)	49.06 (12.75)	0.04
Education (%)				<0.001
*Primary*	358 (49.1)	80 (33.2)	278 (57.0)	
*Secondary*	265 (36.4)	104 (43.2)	161 (33.0)	
*Higher*	106 (14.5)	57 (23.7)	49 (10.0)	
Marital status (%)				<0.001
*Married*	632 (85.2)	226 (91.9)	406 (81.9)	
*Single/Widowed/Divorced*	110 (14.8)	20 (8.1)	90 (18.1)	
Smoking (%)				<0.001
*Current*	46 (6.3)	41 (17.0)	5 (1.0)	
*Ex/Never*	680 (93.7)	200 (83.0)	480 (99.0)	
MET-h/wk	22.1 (7.7–58.1)	23.3 (7.9–63.1)	20.3 (7.6–57.5)	0.80
WHR	0.9 (0.1)	1.0 (0.1)	0.9 (0.1)	<0.001
WC (cm)	95.3 (11.6)	98.1 (10.2)	93.9 (12.0)	<0.001
BMI (kg/m^2^)	29.1 (4.7)	27.8 (4.4)	29.7 (4.8)	<0.001
TG (mmol/L)	1.8 (1.3–2.5)	1.9 (1.3–2.7)	1.8 (1.2–2.4)	0.10
Total cholesterol (mmol/L)	5.2 (1.0)	5.1 (0.9)	5.3 (1.0)	<0.001
HDL-C (mmol/L)	1.1 (0.3)	1.0 (0.2)	1.1 (0.3)	<0.001
LDL-C (mmol/L)	3.3 (0.8)	3.2 (0.8)	3.3 (0.9)	0.11
FBS (mmol/L)	5.7 (1.9)	5.8 (1.8)	5.7 (2.0)	0.32
BS2hr (mmol/L)	6.5 (2.7)	6.2 (2.5)	6.6 (2.8)	0.07
SBP (mmHg)	120.2 (19.0)	124.0 (16.7)	118.3 (19.8)	<0.001
DBP (mmHg)	75.1 (10.1)	77.0 (10.0)	74.2 (10.1)	<0.001
Menopause (%)	-	-	165 (34.3)	-
Pre-diabetes (%)	150 (20.7)	51 (21.2)	99 (20.5)	0.80
Metabolic Syndrome (%)				
WHO	302 (41.8)	107 (44.2)	195 (40.6)	0.36
^a^EGIR	149 (25.6)	49 (25.8)	100 (25.5)	0.94
^a^AACE	179 (30.7)	62 (33.2)	117 (29.5)	0.37

Data represented as mean (SD) for continuous variables with normal distribution and median (25 percentile-75 percentile) for continuous variables with non-normal distribution or n (%) for categorically distributed variables

P-values according to independent sample *t*-test, *χ*2-test and Mann–Whitney test between genders

*BMI*, body mass index; *WC*, waist circumference; *IGT*, impaired glucose tolerance; *IFG*, impaired fasting glucose; *HDL*, high density lipoprotein; *WHR*, waist to hip ratio; *WHO*, World Health Organization; *EGIR*, European Group for the study of Insulin Resistance; *AACE*, American Association of Clinical Endocrinology

^a^ The number of study participants for EGIR and AACE definitions is 590

Both in the physical and mental domains, more men had higher scores in all subscales of SF-36 indicating better HRQoL; however the score of role emotional subscale was higher in women by all three definitions. Among the subscales, physical and social functioning got the highest scores in both men and women respectively according to all three definitions (Table 3).

**Table 3:** Scores of the 36-item Short Form health survey (SF-36), according to different definitions of the metabolic syndrome in men and women

	WHO	EGIR	AACE
Men			
Physical functioning	79.4 (45.3)	79.2 (47.3)	76.5 (45.8)**
Role physical	68.3 (83.1)	69.2 (89.2)	62.3 (87.5)
Bodily pain	73.2 (45.9)	73.2 (49.5)	69.6 (48.7)
General health	66.4 (49.1)	65.0 (52.2)	64.4 (51.0)
Vitality	68.9 (47.1)	69.7 (51.4)	68.9 (50.3)
Social functioning	77.5 (59.9)	72.7 (66.4)	75.1 (65.2)
Role emotional	58.2 (102.4)	48.5 (107.1)	53.5 (104.9)
Mental health	73.1 (46.7)	71.3 (52.1)	74.7 (51.1)
PCS	71.8 (41.9)	71.6 (43.5)	68.2 (42.4)
MCS	69.4 (50.3)	65.6 (53.7)	68.1 (52.6)
Women			
Physical functioning	76.7 (46.4)	76.4 (53.0)	72.9 (49.5)*
Role physical	63.2 (75.8)	69.2 (86.8)	68.1 (81.3)
Bodily pain	70.3 (43.2)	69.6 (48.5)	70.1 (45.2)
General health	64.5 (39.5)	65.9 (44.5)	64.3 (41.7)
Vitality	59.8 (43.4)	60.7 (50.3)	58.7 (46.8)
Social functioning	74.4 (49.0)	76.2 (56.1)	72.4 (52.3)
Role emotional	65.9 (81.2)	66.8 (93.4)	67.4 (86.8)
Mental health	66.7 (41.0)	65.8 (47.2)	64.1 (43.6)
PCS	68.7 (38.6)	70.3 (44.1)	68.9 (41.3)
MCS	66.7 (42.1)	67.4 (48.3)	65.6 (45.2)

Data represented as mean (SD)

*WHO*, World Health Organization; *EGIR*, European Group for the study of Insulin Resistance; *AACE*, American Association of Clinical Endocrinology; *PCS*, physical component summary; *MCS*, mental component summary

* 0.05 ≤ p ≤ 0.07 for poor health-related quality of life which is not significant in α = 0.05 but is significant in α = 0.10

** P < 0.05 for poor health-related quality of life

Table 4 shows the risk of being in the lowest tertile of PCS and MSC according to MetS status by each three definitions. There was no remarkable difference between the rates of poor HRQoL in any of the definitions or either gender. Unadjusted odds ratios (95 % CI) for poor physical health for the WHO, EGIR and AACE definitions were 1.89 (1.00–3.57), 2.04 (0.83–5.03), 1.75 (0.82–3.71) for men respectively, while, in women, they were 1.51 (0.97–2.37), 1.21 (0.67–2.19), 1.56 (0.92–2.64) respectively, significant only in men based on the WHO definition (Table 4).

**Table 4:** Odds ratio (95 % CI) and area under the curve (AUC) for poor physical and mental HRQoL according to different definitions of metabolic syndrome

	WHO	EGIR	AACE
	Physical component summary (PCS)		
Men			
Number of individuals with MetS and poor HRQoL(%)	37 (34.6)	16 (32.7)	24 (38.7)
Adjusted Odds ratio (95 % CI)			
Model 1	1.89 (1.00–3.57)**	2.04 (0.83–5.03)	1.75 (0.82–3.71)
Model 2	1.83 (0.96–3.49)*	2.05 (0.83–5.07)	1.72 (0.77–3.84)
Model 3	1.72 (0.88–3.35)	1.80 (0.69–4.69)	1.95 (0.84–4.53)
AUC	0.64 (0.56–0.73)	0.66 (0.56–0.75)	0.66 (0.56–0.76)
Women			
Number of individuals with MetS and poor HRQoL	70 (35.9)	30 (30)	46 (39.3)
Adjusted Odds ratio (95 % CI)			
Model 1	1.51 (0.97–2.37)*	1.21 (0.67–2.19)	1.56 (0.92–2.64)
Model 2	1.08 (0.67–1.75)	1.06 (0.57–1.96)	0.98 (0.55–1.76)
Model 3	0.96 (0.57–1.60)	0.93 (0.48–1.81)	1.01 (0.55–1.85)
AUC	0.73 (0.68–0.79)	0.74 (0.67–0.80)	0.73 (0.67–0.79)
	Mental component summary (MCS)		
Men			
Number of individuals with MetS and poor HRQoL(%)	33 (30.8)	17 (34.7)	18 (29)
Adjusted Odds ratio (95 % CI)			
Model 1	0.92 (0.49–1.73)	1.16 (0.52–2.59)	0.74 (0.35–1.58)
Model 2	0.95 (0.50–1.80)	1.17 (0.52–2.62)	0.80 (0.36–1.81)
Model 3	0.75 (0.37–1.49)	0.93 (0.38–2.23)	0.97 (0.41–2.28)
AUC	0.63 (0.54–0.71)	0.65 (0.55–0.75)	0.65 (0.55–0.74)
Women			
Number of individuals with MetS and poor HRQoL(%)	71 (36.4)	36 (36)	43 (36.8)
Adjusted Odds ratio (95 % CI)			
Model 1	1.14 (0.73–1.77)	1.15 (0.66–1.99)	1.25 (0.74–2.12)
Model 2	1.00 (0.63–1.58)	1.07 (0.61–1.88)	1.01 (0.57–1.77)
Model 3	0.89 (0.55–1.45)	0.97 (0.54–1.74)	1.00 (0.56–1.79)
AUC	0.65 (0.59–0.71)	0.66 (0.59–0.73)	0.65 (0.59–0.72)

*WHO*, World Health Organization; *EGIR*, European Group for the study of Insulin Resistance; *AACE*, American Association of Clinical Endocrinology; *HRQoL*, Health related quality of life

Model 1: Unadjusted, Model 2: Adjusted for age, Model 3: Adjusted for age, smoking (only in men) (Ref: Never or ex-smoking), education (Ref: Above high school education), marital status (Ref: Married) and menopause (Ref: reproductive age)

* 0.05 ≤ p ≤ 0.07 for poor health-related quality of life

** *P* < 0.05 for poor health-related quality of life

Compared with women, after adjustment for confounding variables, in physical HRQoL all definitions showed higher odds ratios in men as follows: WHO 1.72 (95 % CI; 0.88–3.35) vs. 0.96 (95 % CI; 0.57–1.60), EGIR 1.80 (95 % CI; 0.69–4.96) vs. 0.93 (95 % CI; 0.48–1.81), AACE 1.95 (95 % CI; 0.84–4.53) vs. 1.01 (95 % CI; 0.55–1.85); however, in mental HRQoL women had higher odds ratios than men. Although only men with WHO-defined MetS had significant odds ratio for reporting poor physical HRQoL, after adjustment for confounding factors the AACE definition had higher odds ratio for reporting poor physical HRQoL in both genders. In this definition, in physical HRQoL, the odds ratio increased after controlling confounding factors (Table 4).

After adjustment for confounding variables, ROC analysis showed no significant superiority in the discriminatory powers of the three different MetS definitions in detecting poor HRQoL in physical or mental domains in either gender. However, especially in physical health, women showed higher AUCs for all definitions, in comparison to men (Table 4).

## Discussion

In the current study, after adjusting confounding variables, none of the three IR-based definitions of MetS could significantly detect poor HRQoL in the physical or mental domains. Moreover ROC analysis showed no significant superior diagnostic value for any of the three definitions; however the WHO definition identified more patients with MetS (41.8 %) than the other IR-based definitions. Only the WHO definition of MetS significantly detected poor physical quality of life in men before adjustment for potential confounders.

Although previous studies have documented the predictive value of different WC-based definitions of MetS on the objective and subjective outcomes of CVD and HRQoL [13, 15], to the best of our knowledge, this is the first report comparing the diagnostic values of the IR-based definitions of this syndrome in detecting poor HRQoL. Findings from studies investigating the impact of MetS on the risk of CVD, as a measurable outcome, show both similarities and differences. Based on previous findings, compared to the IR-based definitions, the WC-based definitions of MetS showed a superior predictive value of non- fatal CVD among an elderly Dutch population [13]; Consistent to this, another study showed that WHO and EGIR definitions were associated with lower risk of all cause CVD than WC-based definitions in men enrolled in a multiethnic study [24]. Moreover, a study conducted in Turkey, reported that WC-based definitions of MetS were more useful than IR - based definitions in showing increased Framingham risk of cardiovascular disease [25], results consistent with those of our previous study, showing that most of the WC-based definitions had a significant association with poor HRQoL [15]. It seems that central adiposity, the stem of WC-based definitions, plays the main role in MetS objective and subjective outcomes [26].

Can et al. showed that, in an adult Turkish population the AACE definition of MetS identified more individuals at increased cardio-metabolic risk than the WHO or EGIR definitions, a result consistent with that of our study showing the highest odds ratio of having poor HRQoL in the AACE definitions compared to WHO and EGIR definitions; however it should be considered that in the Turkish sample, the AACE definition detected more patients having MetS than the WHO and EGIR definitions, which is contrary to our findings, in which the WHO definition detects the highest rate of individuals with MetS [25].

Although in our previous study, the association between poor physical HRQoL and different WC-based definitions of MetS was significant in women but not in men [14], in the current study it is interesting to note that the odds of having poor physical HRQoL is lower in women than in men, a sex specific difference which may be associated to the use of IR as the main component of MetS definitions used in this study; in this regard, a study by Schlotz et al. assessing the relation between IR and HRQoL, showed a significant association between poor HRQoL and some subscales of PCS in men but not women after controlling for confounding variables [16]. Based on the Masharani et al. findings, this sex specific difference in the association between IR-based definitions of MetS with HRQoL may be due to sex differences in the associations between insulin resistance, regional adipose stores, and lipids values which may result in higher prevalence of obesity and high WC in men in association with IR [27]; this is while previous studies revealed that abdominal obesity is the major component of association between MetS and poor HRQoL in women [28]. On the other hand, in type 2 diabetic patients, which is one of the main manifestations of IR, women compared to men, showed worse quality of life and mental well-being [29].

Based on our findings, the IR-based definitions of MetS were not associated with the mental HRQoL in any of the three definitions, results consistent with those of our previous study in which WC-based definitions of MetS showed no significant association with poor mental HRQoL in adult Tehranians [15]. Moreover previous studies have shown that IR and its related measures are associated with poor HRQoL in the domains of physical health but not in domains of mental health [16]. The association between IR and depression has been shown in some previous studies, including a recent meta-analysis, in which a small, but significant association was observed between IR and depression [30], an observation not found in our study.

Based on our knowledge, this is the first report comparing the diagnostic impact of different IR based definitions of the metabolic syndrome in a large sample of adults in the general population. Our study has some limitations; first, this study was conducted using a cross - sectional design, so we are unable to draw conclusions regarding the causal association between MetS and HRQoL. Second, there may be yet other confounding factors such as depression and economic status that affect HRQoL, factors that we did not adjust for. Third, since the three definitions of MetS tended to be associated with poor physical HRQoL in men, the lack of significant statistical results could be related to low statistical power. Moreover, microalbuminuria, which is included in the WHO definition, was omitted from our study because of lack of data. Finally in the AACE definition, due to inadequate information, we did not include acanthosis nigricans, polycystic ovary syndrome, nonalcoholic fatty liver disease, non-Caucasian ethnicity and sedentary life style in the criteria needed for an individual to be considered at high risk of being insulin resistant.

## Conclusions

Although the AACE definition had a higher odds of having poor physical HRQoL compared to the WHO and EGIR definitions of MetS, none the three definitions could detect poor HRQoL after adjusting potential confounders in either physical or mental domains in men and women. Accordingly, ROC analysis failed to show any significant superiority in the discriminatory power of the different definitions over each other.

## Abbreviations

AACE: American Association of Clinical Endocrinology
ADA: American Diabetes Association
AHA/NHLBI: American Heart Association/National Heart, Lung, and Blood Institute
AUC: Area Under the Curve
BMI: Body Mass Index
CHD: Coronary Heart Disease
CVD: Cardiovascular Disease
CI: Confidence Interval
EGIR: European Group for the study of Insulin Resistance
HRQoL: Health-Related Quality of Life
HDL-C: High Density Lipoprotein-Cholesterol
IDF: International Diabetes Federation
IR: Insulin Resistance
JIS: Joint Interim Statement
MetS: Metabolic Syndrome
MCS: Mental Component Summary
NCEP-ATP III: National Cholesterol Education Program - Adult Treatment Panel III
OR: Odds Ratio
PCS: Physical Component Summary
ROC: Receiver Operating Characteristic
SF-36: Short Form Health survey
TG: Triglyceride
WC: Waist Circumference
WHO: World Health Organization
WHR: Waist to Hip Ratio
